# Supplementary material for: Wnt-3a Induces Cytokine Release in Human Mast Cells
Source: Cells. 2019 Nov 1;8(11):1372. doi: 10.3390/cells8111372 (PMC6912728; doi:10.3390/cells8111372)
Supplement: Supplementary file 1 [file cells-08-01372-s001.pdf]

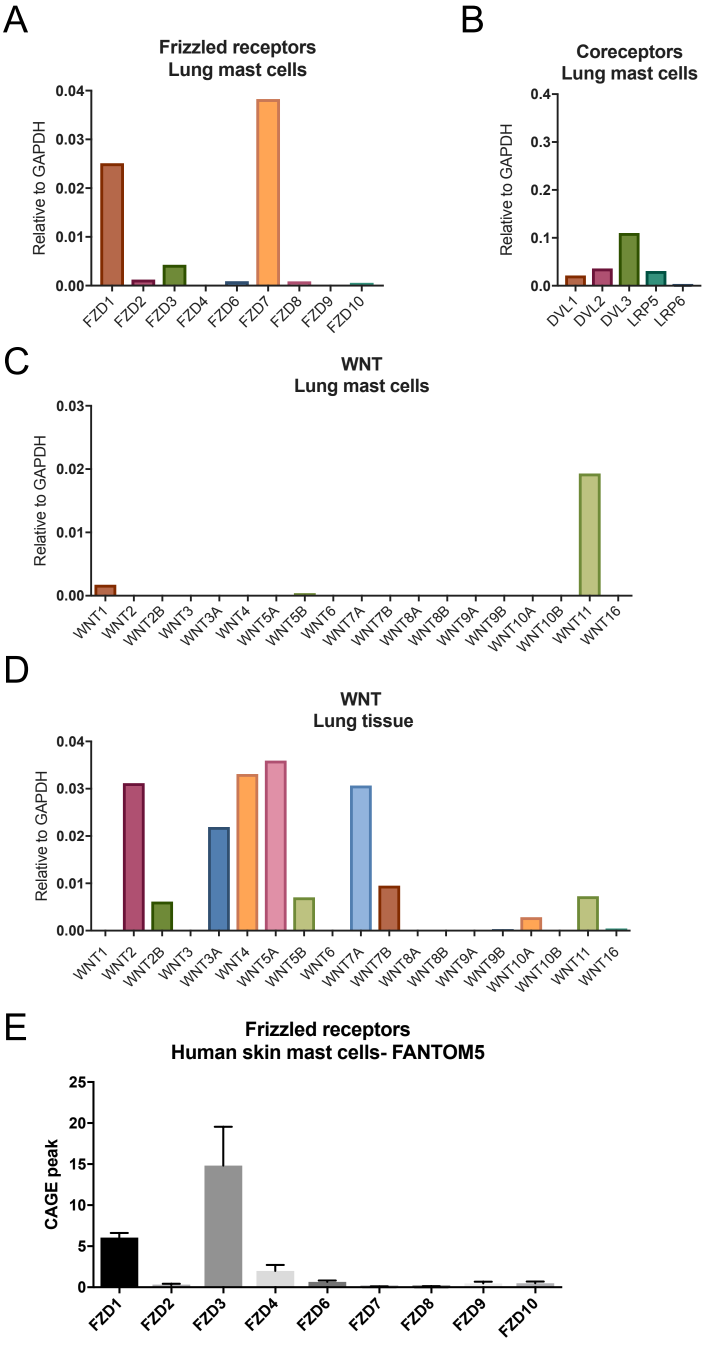

Supplementary Figure 1. mRNA expression of components of the Wnt signaling system in human mast cells

mRNA was extracted from sorted human human lung mast cells (A-C) and human lung tissue and qPCR was performed for frizzled receptors (A), DVL1-3 and LRP5/6 (B), and all 19 Wnts (C-D) using a Human WNT Pathway TaqMan Array. mRNA was pooled from 3 individual donors. FZD expression in human skin mast cells was extrapolated from the FANTOM5 online depository, mean with SEM, n=4 (E).

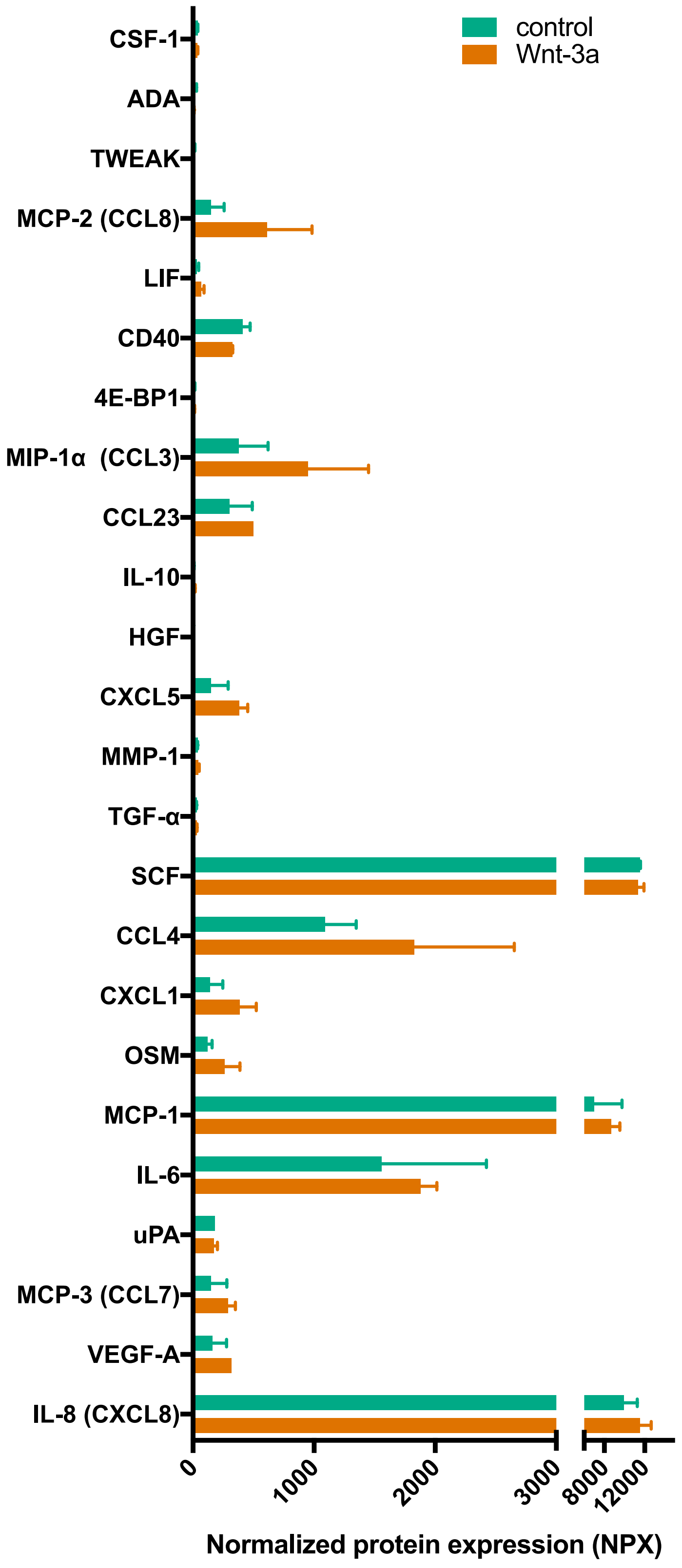

Supplementary Figure 2. Olink screen of released cytokines

CBMCs were stimulated with Wnt-3a for 24 hours and the supernatent was run on Olinks proteomics inflammation panel. Proteins with a mean NPX value greater then 10 is shown. n=2.
